# Pericardial abscess: A rare complication of disseminated melioidosis in a patient with relapsed multiple myeloma

**DOI:** 10.1590/0037-8682-0018-2025

**Published:** 2025-03-31

**Authors:** Chee Yik Chang, Kai Xin Ang

**Affiliations:** 1 Hospital Sultanah Aminah, Medical Department, Infectious Diseases Unit, Johor Bahru, Johor, Malaysia.; 2 Hospital Sultanah Aminah, Medical Department, Johor Bahru, Johor, Malaysia.

**FIGURE 1:**
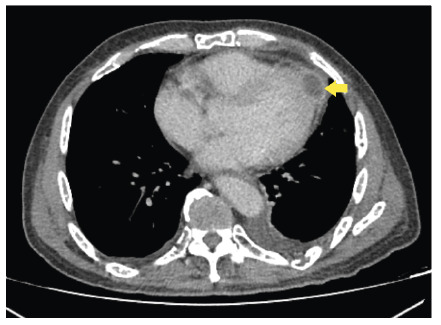
Computed tomography of the thorax showing a pericardial rim-enhancing collection measuring 2.6 x 1.9 x 1.6 cm.

A 58-year-old man with multiple myeloma who was in remission following chemotherapy and autologous stem cell transplantation relapsed in 2021. He developed numerous painful swelling and fever after undergoing salvage chemotherapy. Blood cultures revealed the presence of *Burkholderia pseudomallei*. Computed tomography revealed an abscess in the pericardium (Figure 1), chest walls, and axillae. Intravenous administration of meropenem was commenced; however, drainage was not possible owing to severe thrombocytopenia. The patient’s condition improved after 4 weeks of antibiotic therapy, and he was discharged with oral amoxicillin-clavulanic acid. The administration of trimethoprim-sulfamethoxazole was avoided owing to cytopenia.

Melioidosis, caused by the gram-negative bacterium *B. pseudomallei*, presents with diverse manifestations such as pneumonia, septicemia, and internal organ abscesses^1^. Disseminated disease is particularly common in immunocompromised individuals such as those with diabetes mellitus and hematologic malignancies. The immunosuppressed state facilitates the hematogenous spread of the pathogen, resulting in the development of more severe forms of the disease^2^. Cardiac involvement in melioidosis, although rare, can manifest as myocarditis, endocarditis, and pericarditis. Myocarditis often presents as arrhythmia or heart failure. Endocarditis may involve native or prosthetic valves, leading to valvular destruction and embolic phenomena. Pericardial involvement, which is also rare, can range from acute pericarditis to pericardial effusion; pericardial abscess formation may be observed in extremely rare cases^3^.
